# Interleukin-3 is a predictive marker for severity and outcome during SARS-CoV-2 infections

**DOI:** 10.1038/s41467-021-21310-4

**Published:** 2021-02-18

**Authors:** Alan Bénard, Anne Jacobsen, Maximilian Brunner, Christian Krautz, Bettina Klösch, Izabela Swierzy, Elisabeth Naschberger, Malgorzata J. Podolska, Dina Kouhestani, Paul David, Torsten Birkholz, Ixchel Castellanos, Denis Trufa, Horia Sirbu, Marcel Vetter, Andreas E. Kremer, Kai Hildner, Andreas Hecker, Fabian Edinger, Matthias Tenbusch, Petra Mühl-Zürbes, Alexander Steinkasserer, Enrico Richter, Hendrik Streeck, Marc M. Berger, Thorsten Brenner, Markus A. Weigand, Filip K. Swirski, Georg Schett, Robert Grützmann, Georg F. Weber

**Affiliations:** 1 Department of Surgery, Friedrich-Alexander University (FAU) Erlangen-Nürnberg and Universitätsklinikum Erlangen, Erlangen, Germany; 2 Department of Anesthesiology, Friedrich-Alexander University (FAU) Erlangen-Nürnberg and Universitätsklinikum Erlangen, Erlangen, Germany; 3 Department of Thoracic Surgery, Friedrich-Alexander University (FAU) Erlangen-Nürnberg and Universitätsklinikum Erlangen, Erlangen, Germany; 4 Department of Internal Medicine 1 - Gastroenterology, Pneumology and Endocrinology, Friedrich-Alexander University (FAU) Erlangen-Nürnberg and Universitätsklinikum Erlangen, Erlangen, Germany; 5 Department of Surgery, University Hospital, Giessen, Germany; 6 Institute of Clinical and Molecular Virology, Friedrich-Alexander University (FAU) Erlangen-Nürnberg and Universitätsklinikum Erlangen, Erlangen, Germany; 7 Department of Immune Modulation, Friedrich-Alexander University (FAU) Erlangen-Nürnberg and Universitätsklinikum Erlangen, Erlangen, Germany; 8 Institute of Virology, University Hospital, Bonn, Germany; 9 Department of Anesthesiology and Intensive Care Medicine, University Hospital Essen, University Duisburg-Essen, Essen, Germany; 10 Department of Anesthesiology, Heidelberg University Hospital, Heidelberg, Germany; 11 Center for Systems Biology, Massachusetts General Hospital and Harvard Medical School, Boston, MA USA; 12 Department of Internal Medicine 3 - Rheumatology and Immunology, Friedrich-Alexander University (FAU) Erlangen-Nürnberg and Universitätsklinikum Erlangen, Erlangen, Germany

**Keywords:** Viral infection, Risk factors

## Abstract

Severe acute respiratory syndrome coronavirus 2 (SARS-CoV-2) is a worldwide health threat. In a prospective multicentric study, we identify IL-3 as an independent prognostic marker for the outcome during SARS-CoV-2 infections. Specifically, low plasma IL-3 levels is associated with increased severity, viral load, and mortality during SARS-CoV-2 infections. Patients with severe COVID-19 exhibit also reduced circulating plasmacytoid dendritic cells (pDCs) and low plasma IFNα and IFNλ levels when compared to non-severe COVID-19 patients. In a mouse model of pulmonary HSV-1 infection, treatment with recombinant IL-3 reduces viral load and mortality. Mechanistically, IL-3 increases innate antiviral immunity by promoting the recruitment of circulating pDCs into the airways by stimulating CXCL12 secretion from pulmonary CD123^+^ epithelial cells, both, in mice and in COVID-19 negative patients exhibiting pulmonary diseases. This study identifies IL-3 as a predictive disease marker for SARS-CoV-2 infections and as a potential therapeutic target for pulmunory viral infections.

## Abstract (web summary)

Here, the authors identify interleukin-3 as a predictive marker for severity and outcome of SARS-CoV-2 infection in a multi-center, prospective study and find that patients with severe COVID-19 have reduced circulating plasmacytoid dendritic cell levels compared to non-severe COVID-19 patients.

## Introduction

The SARS-CoV-2 infection has spread worldwide^1^. Patients can be asymptomatic or present with a myriad of symptoms, including dry cough, fever, and fatigue^2^. Eventually, SARS-CoV-2 infections may progress to acute respiratory distress syndrome (ARDS) and lung injury, which is associated with high morbidity and mortality^3^. To date, treatment options for patients with severe cases of coronavirus disease 2019 (COVID-19) are limited, so far leading to over 1.6 million deaths worldwide (WHO statistics). It is therefore a health priority to identify patients at risk in early stages of the disease, to better understand the underlying immunological mechanisms leading to disease severity and explore new therapeutic options.

SARS-CoV-2 infects cells expressing the surface receptors ACE2 and TMPRSS2^4^. The active replication of the virus may result in the pyroptosis of infected cells leading to the release of inflammatory signals^5^. The recognition of these signals by adjacent cells (epithelial cells or alveolar macrophages) triggers the secretion of inflammatory cytokines and chemokines in the micro-environment resulting in the recruitment of antiviral innate and adaptive immune cells^6^. Several studies have indicated that the impaired innate antiviral defences against SARS-CoV-2 coupled with the hyperinflammatory immune response to the virus is a major driver of disease severity and mortality^7,8^. Indeed, patients with severe SARS-CoV-2 infections exhibit high levels of circulating inflammatory cytokines and increased T cell and monocyte recruitment into the lungs^9^, which may cause lung damage and the cytokine release syndrome. For this reason, immunosuppressive therapeutic agents such as corticosteroids and IL-6 antagonists are being clinically tested to eventually reduce the hyperinflammation-mediated organ damage^6^.

Interleukin-3, a hematopoietic growth factor produced by T cells^10^ and in a lesser extend by mast cells^11^, eosinophils^12^, and innate response activator B cells^13^, was described to play a key role during inflammatory diseases. For instance, we recently identified that IL-3 amplified acute inflammation in sepsis by fuelling the cytokine storm, with high levels of IL-3 predicting death^13^. IL-3 has also been shown to induce experimental autoimmune encephalitis (EAE) by promoting leukocyte migration into the brain^14^. In contrast, IL-3 reduces the severity of collagen-induced arthritis by modulating the development of Foxp3 regulatory T cells^15^. The function of IL-3 during viral immunity, however, remains rudimentary.

Here we report that IL-3 may be an independent prognostic marker for the outcome of severe SARS-CoV-2 infections. Low plasma IL-3 levels are associated with increased disease severity as well as increased viral load and mortality in SARS-CoV-2 infected patients. Likewise, mice treated with recombinant IL-3 had reduced mortality, viral load, and loss of weight upon lethal pulmonary viral infection. Mechanistically, we observed that IL-3 improved innate viral immunity by promoting the recruitment of antiviral plasmacytoid dendritic cells (pDCs) into the airways and that IL-3 promoted pDC recruitment into the lungs by stimulating the secretion of CXCL12 from pulmonary epithelial cells.

## Results and Discussion

**Fig. 1:**
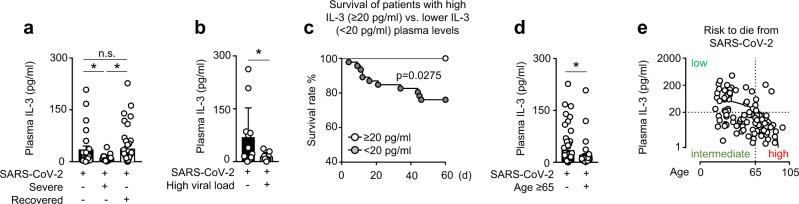
Low plasma interleukin-3 levels are associated with severity and outcome in COVID-19. **a** Plasma IL-3 levels of SARS-CoV-2^+^ patients with or without severe disease and in patients that had recovered from infection. *p* = 0.0468 for non-severe vs severe; *p* = 0.0016 for severe vs recovered; and *p* = 0.8441 for non-severe vs recovered. *n* = 92. **b** Plasma IL-3 levels of SARS-CoV-2^+^ patients with or without high viral load. *n* = 21. **c** Kaplan–Meier analysis showing the survival of SARS-CoV-2^+^ patients with high (≥20 pg/ml) or low (<20 pg/ml) plasma IL-3 levels (measured within 24 h after admission). *n* = 64. **d** Plasma IL-3 levels of SARS-CoV-2^+^ patients older or younger than 65 years. *n* = 92. **e** Analysis of plasma IL-3 levels (20 pg/ml identified by minimal *p*-value approach) and age defining risk groups to die (low and intermediate vs high; OR: 14.091; 95% CI: 1.680–118.218). *n* = 110. Data are mean ± S.E.M., *P < 0.05, **P < 0.01, ***P < 0.001, unpaired, two-tailed Student’s *t* test using Welch’s correction for unequal variances or Mann–Whitney test were used. Source data are provided as a Source Data file.

Patients with severe COVID-19, characterized by necessity for intensive care treatment and high plasma CRP levels (Supplementary Fig. 1a), had reduced plasma IL-3 levels as compared to patients with non-severe COVID-19 or patients that had recovered (Fig. 1a). As well, patients with high viral load presented lower plasma IL-3 levels as compared to patients with low viral load (Fig. 1b), both results suggesting that plasma IL-3 levels were associated with disease severity in SARS-CoV-2 infections and that IL-3 does not seem to fuel the cytokine storm. In this prospective multicentric observation study, Kaplan–Meier survival analysis revealed that plasma IL-3 levels may influence the outcome of SARS-CoV-2 infections: using a minimal *p*-value approach, patients with plasma IL-3 levels <20 pg/ml at admission had a poorer prognosis as compared to patients with plasma IL-3 levels ≥20 pg/ml at admission (Fig. 1c and Supplementary Table 1), this association remaining significant after adjusting for prognostic parameters in multivariate analysis (Supplementary Table 2). Older age was described to be associated with greater risk to develop severe COVID-19^16^. Patients older than 65 years showed reduced plasma IL-3 levels as compared to patients younger than 65 years (Fig. 1d). Thus, the analysis of plasma IL-3 levels and patient age allowed to identify three groups at risk to die from COVID-19: patients <65 years with plasma IL-3 levels ≥20 or <20 pg/ml had a low to intermediate risk to die whereas patients ≥65 years with plasma IL-3 levels <20 pg/ml had a high risk to die (OR: 14.091; 95% CI: 1.680 – 118.218) (Fig. 1e, Supplementary Fig. 1b and Supplementary Table 3). Collectively, our results suggest that IL-3 may be an early predictive marker to identify patients at risk during SARS-CoV-2 infections.

**Fig. 2:**
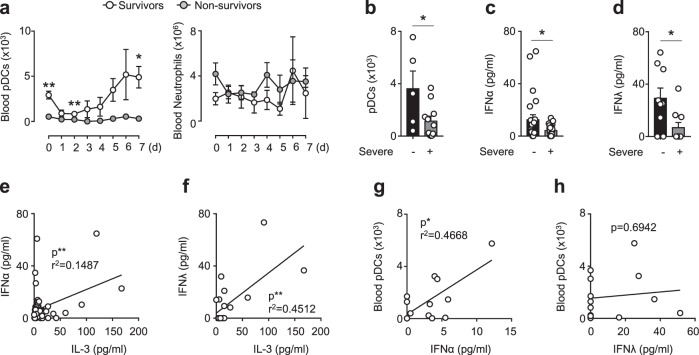
Plasma type I interferon levels are associated with plasma interleukin-3 levels and circulating pDCs in COVID-19. **a** Absolute numbers of circulating pDCs and neutrophils in SARS-CoV-2^+^ patients from their admission to ICU and 1 to 7 days later. *p* = 0.0018 (pDCs d0), *p* = 0,0061 (pDCs d2), and *p* = 0.0227 (pDCs d7). *n* = 9. **b**–**d** Absolute numbers of circulating pDCs (*n* = 16; *p* = 0.0331) (**b**) and plasma IFNα (*n* = 48, *p* = 0.0459) (**c**), and IFNλ (*n* = 19, *p* = 0.0167) (**d**) levels of patients with severe or non-severe COVID-19 infections. *n* = 16–48. **e**–**f** Correlation between plasma IL-3 levels and plasma IFNα (**e**) and IFNλ (**f**) levels in SARS-CoV-2^+^ patients. *n* = 20–47. **g**–**h** Correlation between absolute numbers of circulating pDCs and plasma IFNα (**g**) and IFNλ (**h**) levels in SARS-CoV-2^+^ patients. *n* = 12–13. Data are mean ± S.E.M., *P < 0.05, **P < 0.01, ***P < 0.001, unpaired, two-tailed Student’s *t* test using Welch’s correction for unequal variances was used. Source data are provided as a Source Data file.

We next investigated whether IL-3 might protect against viral infection. Non-survivors from severe SARS-CoV-2 infection displayed lower circulating pDC numbers over time as compared to survivors whereas no differences could be detected in the numbers of circulating neutrophils (Fig. 2a) suggesting a protective effect of pDCs during SARS-CoV-2 infections. Reduced numbers of circulating pDCs as well as reduced plasma IFNα and IFNλ levels were also observed in patients with severe COVID-19 as compared to patients with non-severe COVID-19 (Fig. 2b–d). Moreover, plasma IL-3 levels were associated to plasma IFNα and IFNλ levels in SARS-CoV-2^+^ patients (Fig. 2e, f) and plasma IFNα levels correlated with the number of circulating pDCs (Fig. 2g). However, the numbers of pDCs were not associated to plasma IFNλ levels in COVID-19 patients (Fig. 2h). These data suggest that IL-3 may protect against SARS-CoV-2 infections by increasing the amount of antiviral pDCs.

**Fig. 3:**
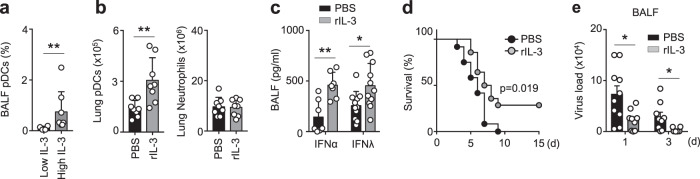
Interleukin-3 protects against viral infection by increasing pDC numbers in the lungs. **a** Percentage of pDCs in BALF of patients with pulmonary diseases with high or low BALF IL-3 levels. *p* = 0.004. Mann–Whitney test. *n* = 12. **b** Absolute numbers of pDCs and neutrophils in the lungs of naive mice 24 h after the i.n. injection of PBS or rIL-3, followed by an i.n. injection of CpG 8 h later. *p* = 0.0091. *n* = 8. **c** Levels of IFNα and IFNλ in the BALF of naive mice that received an i.n. injection of PBS or rIL-3, followed by an i.n. injection of CpG 8 h later, and were sacrificed 16 h later. *n* = 14 for IFNα (*p* = 0.0205) and *n* = 22 for IFNλ (*p* = 0.018). **d**, **e** Survival (**d**) and viral load in the lungs (**e**) of naive WT mice after i.n. infection with 6 × 10^6^ PFU of HSV-1. Mice received an i.n. injection of PBS or rIL-3 just before HSV-1 infection. *n* = 26 (d) and *n* = 19 for d1 (*p* = 0.0151) and *n* = 15 for d3 (*p* = 0.0439) (e). Data are mean ± S.E.M., *P < 0.05, **P < 0.01, ***P < 0.001, unpaired, two-tailed Student’s *t* test using Welch’s correction for unequal variances was used. Source data are provided as a Source Data file.

To further assess this possible mechanism, we conducted bronchoalveolar lavage fluid (BALF) analysis in COVID-19 negative (COVID-19−) patients exhibiting pulmonary diseases (Supplementary Table 4). Although these samples might not perfectly reflect the immune environment occuring in BALF during SARS-CoV-2 infections, they allowed us to investigate the link between IL-3 and pDCs in BALF in the context of pulmonary inflammation. Our analysis revealed that (i) patients with high BALF IL-3 levels showed increased percentages of pDCs as compared to those with low BALF IL-3 levels (Fig. 3a); (ii) plasma IL-3 levels tended to positively correlate with BALF IL-3 levels (Supplementary Fig. 2a); and (iii) the percentage of circulating pDCs were correlated to the percentage of BALF pDCs (Supplementary Fig. 3b). In mice, intranasal (i.n.) IL-3 administration increased the number of pDCs, but not neutrophils, in the lung parenchyma as compared to controls (Fig. 3b and Supplementary Fig. 3a) and enhanced the secretion of IFNα and IFNλ in the BALF after subsequent i.n. CpG injection (Fig. 3c). Depletion of pDCs induced a strong reduction of IFNα levels in BALF of mice pre-treated with IL-3 upon i.n. CpG administration, whereas IFNλ secretion was only partially impaired (Supplementary Fig. 3b, c). To assess if IL-3 protects during viral pneumonia, we intranasally infected mice with Herpes Simplex Virus-1 (HSV-1), a virus described to be the most frequently isolated pathogen in the lungs of patients with severe respiratory distress^17^ and being related to poor outcome in critically ill patients^18^. Compared to controls, naive WT mice pre-treated with IL-3 and subjected to lethal HSV-1 infection displayed a lower mortality rate (Fig. 3d). Moreover, IL-3 pre-treatment before HSV-1 infection resulted in increased viral clearance in the BALF (Fig. 3e) that was accompanied with reduced weight loss and improved body temperature (Supplementary Fig. 3d, e). Collectively, these data indicate that IL-3 induces antiviral immunity in the lung during viral infection.

**Fig. 4:**
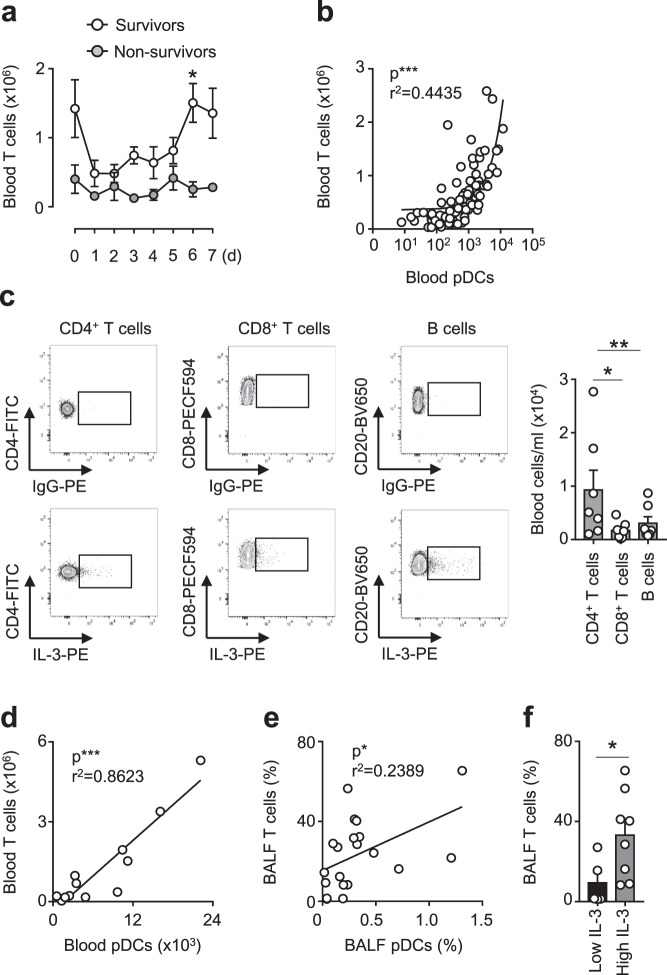
Interleukin-3 is produced by T cells in COVID-19. **a** Absolute numbers of circulating T cells in SARS-CoV-2^+^ patients from their admission to ICU and 1 to 7 days later. *p* = 0.0154 (d6). *n* = 9. **b** Correlation between the absolute numbers of circulating T cells and circulating pDCs in SARS-CoV-2^+^ patients. Pearson *r* test (*p* < 0.0001). *n* = 87. **c** Absolute numbers of circulating CD4^+^ T cells, CD8^+^ T cells, and B cells expressing IL-3 in SARS-CoV-2^+^ patients. *p* = 0.024 for CD4 T cells vs CD8 T cells; *p* = 0.0036 for CD4 T cells vs B cells. *n* = 7. **d** Correlation between the number of circulating T cells and the number of circulating pDCs of patients with pulmonary diseases. Pearson *r* test (*p* < 0.0001). *n* = 12. **e** Correlation between the percentage of BALF T cells and the percentage of BALF pDCs of patients with pulmonary diseases. Pearson *r* test (*p* = 0.0288). *n* = 20. **f** Percentage of T cells in the BALF of patients with pulmonary diseases with high or low IL-3 BALF levels. *p* = 0.0452. *n* = 13. Data are mean ± S.E.M., *P < 0.05, **P < 0.01, ***P < 0.001, unpaired, two-tailed Student’s *t* test using Welch’s correction for unequal variances and paired, two-tailed Student’s *t* test were used. Source data are provided as a Source Data file.

In humans, T cells have been described as a major cellular source of IL-3 during inflammation^10^. We therefore investigated whether T cells are a source of IL-3 during SARS-COV-2 infections. The enumeration of T cells in SARS-COV-2^+^ patients showed that survivors from severe SARS-CoV-2 infections displayed higher circulating T cells over time as compared to non-survivors (Fig. 4a) and that circulating T cells were correlated to circulating pDCs in SARS-COV-2^+^ patients (Fig. 4b). Flow cytometry analysis revealed that IL-3 is produced by CD4^+^ T cells and, to a lesser extend, by CD8^+^ T cells and B cells (Fig. 4c). In COVID-19− patients exhibiting pulmonary diseases, the number of circulating T cells were correlated to the number of circulating pDCs (Fig. 4d). In their BALF, the percentage of T cells were correlated to the percentage of pDCs (Fig. 4e) and high levels of IL-3 were associated with an increased percentage of T cells (Fig. 4f). Altogether, these data indicate that T cells are robust producers of IL-3 in COVID-19 patients during SARS-COV-2 infections and suggest that the reduced plasma IL-3 levels and circulating pDCs observed in patients with severe SARS-CoV-2 infections might reflect the lymphopenic state observed in these patients^19^.

**Fig. 5:**
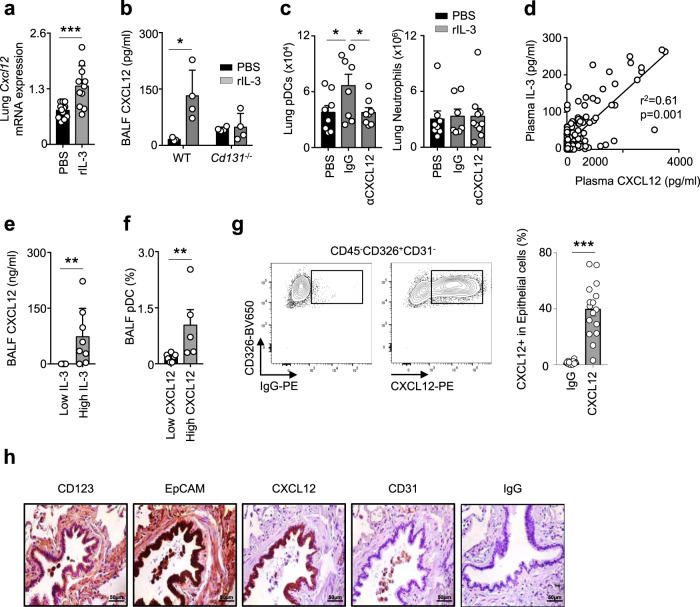
Interleukin-3 promotes the recruitment of pDCs into the lung in a CXCL12-dependent manner. **a** Relative mRNA expression of *Cxcl12* in the lungs of naive mice 24 h after the i.n. injection of PBS or rIL-3. *p* = 0.0009. *n* = 11–12. **b** Level of CXCL12 in the BALF of WT or *Cd131*^*−/−*^ mice 24 h after the i.n. injection of PBS or rIL-3. *n* = 7 for WT (*p* = 0.0416) and *n* = 8 for *Cd131*^*−/−*^ (*p* = 0.8046). **c** Absolute number of pDCs and neutrophils in the lungs of naive mice 24 h after the i.n.injection of PBS, IgG or anti-CXCL12 followed by i.n. injection of PBS (black) or IL-3 (grey). *p* = 0.0371 for IgG vs αCXCL12 and *p* = 0.437 for PBS vs IgG. *n* = 8–9. **d** Correlation between plasma IL-3 and CXCL12 levels of SARS-CoV-2^+^ patients. *n* = 181. **e** Level of CXCL12 in the BALF of patients with pulmonary diseases with high or low IL-3 BALF levels. *p* = 0.0078. *n* = 13. **f** Percentage of pDCs in the BALF of patients with pulmonary diseases with high or low CXCL12 BALF levels. *p* = 0.0051. *n* = 12. **g** Percentage of CXCL12^+^ epithelial cells in the lungs of patients with pulmonary inflammation. *p* < 0.0001. *n* = 16. **h** Immunohistochemistry of CD123, EpCAM, CXCL12, CD31, and IgG in the lungs of patients with pulmonary inflammation. *n* = 3 different lungs. Data are mean ± S.E.M., *P < 0.05, **P < 0.01, ***P < 0.001, two-tailed unpaired or Mann–Whitney test were used. Source data are provided as a Source Data file.

To further characterize the mechanism involved in IL-3-mediated pDC recruitment, we quantified the mRNA expression of chemokines known to drive pDC migration into inflamed tissues^20^. We observed that only *Cxcl12* expression was increased by IL-3 treatment in lungs of mice upon CpG administration (Fig. 5a and Supplementary Fig. 3f). We also detected increased CXCL12 levels in the BALF of WT mice treated with IL-3 (Fig. 5b) and in the supernatant of ex vivo cultured lung cells derived from naive WT mice upon IL-3 stimulation (Supplementary Fig. 3g). The induction of CXCL12 was mediated through the IL-3 receptor common β-chain (CD131), as no increase in CXCL12 levels was observed in the BALF of *Cd131*^*-/-*^ mice upon IL-3 stimulation (Fig. 5b). I.n. injection of CXCL12 in mice resulted in increased numbers of pDCs but not neutrophils in the lungs (Supplementary Fig. 3h). Additionally, i.n. injection of CXCL12-neutralizing antibodies prevented pDC influx into the lungs of WT mice upon IL-3 injection, whereas no difference was observed for neutrophils (Fig. 5c). In SARS-CoV-2^+^ patients, plasma IL-3 levels strongly correlated with plasma CXCL12 levels, but not with plasma IL-6, TNF, and CRP levels (Fig. 5d and Supplementary Fig. 4a–c). CXCL12 plasma levels were also not correlated with circulating pDCs (Supplementary Fig. 4d). In BALF of COVID-19− patients exhibiting pulmonary diseases, IL-3 positively correlated with CXCL12 levels (Fig. 5e) and high levels of CXCL12 were associated with increased percentages of pDCs (Fig. 5f).

We found that only CD45^−^ non-haematopoietic cells expressed the α-chain of the IL-3 receptor (CD123) in the lungs of naive mice (Supplementary Fig. 5a) although we cannot exclude the expression of CD123 in rare CD45^+^ cells. Likewise, only CD45^-^ cells secreted CXCL12 after IL-3 stimulation (Supplementary Fig. 5b). Flow cytometry analyses revealed that only epithelial cells were found to overexpress CXCL12 in the lungs of mice upon ex vivo IL-3 stimulation (Supplementary Fig. 5c). As well, CXCL12 was only expressed by CD326^+^ CD123^+^ epithelial cells in human lungs of COVID-19^-^ patients (Fig. 5g–h and Supplementary Table 5).

Collectively, our study revealed that plasma IL-3 levels might allow risk stratification in patients with SARS-CoV-2 infections. We therefore propose IL-3 as a predictive marker for disease severity and clinical outcome. Based on its ability to improve local antiviral defence mechanisms by recruiting pDCs, recombinant IL-3, or CD123 receptor agonists, may therefore have the potential as novel therapeutic agents in SARS-CoV-2 infected patients.

## Methods

### Animals

Balb/c (WT), C57Bl/6J (WT) (Janvier, Le Genest-Saint-Isle, France), and *Cd131*^*-/-*^ (C57Bl/6J background, bred in-house) female mice were used in this study. Majority of the mice were 8–12 weeks old when sacrificed. All animal protocols were approved by the animal review committee from the University Hospital of Dresden and Erlangen and the local governmental animal committee. Mice were breed in animal facility where i) air exchange rate of up to 30 times per hour is possible in the animal rooms; ii) the animals are subject to a 12 h light/dark rhythm; iii) air humidity is between 45 and 65%; iv) the temperature is 20 and 24 °C; v) the animals receive autoclaved and autoclavable water; and vi) food and water are ad libitum.

### Mouse infection

Naive mice were anesthetized with isoflurane and infected intra-nasally with 8 µg of CpG (Enzo Life Sciences, Farmingdale, NY, USA), 400 ng of recombinant IL-3 (R&D Systems, Minneapolis, MN, USA), 500 ng of recombinant CXCL12 (Peprotech, Rocky Hill, NJ, USA), 50 µg of IgG or anti-CXCL12 (R&D systems), and 6 × 10^6^ PFU HSV-1 in a volume of 15 µl saline. For the pDCs depletion experiment, 150 µg of IgG or anti-CD317 (Miltenyi) were injected intravenously 15 h before IL-3 and CpG injection.

### Murine leukocytes isolation

After lungs harvest, single cell suspensions were obtained as follows: perfused lungs were cut in small pieces and subjected to enzymatic digestion with 450 U/ml collagenase I (Sigma Aldrich), 125 U/ml collagenase IX (Sigma Aldrich), 60 U/ml hyaluronidase (Sigma Aldrich), 60 U/ml Dnase (Sigma Aldrich), and 20 mM Hepes (Thermo Fisher Scientific, Waltham, MA, USA) for 1 h at 37 °C while shaking. Bronchoalveolar lavage (BAL) was performed by flushing the lungs with 2 × 1 ml of PBS to retrieve the infiltrated and resident leukocytes. Total viable cell numbers were obtained using Trypan Blue (Carl Roth).

### Virus preparation and titration

Preparation of HSV-1 stocks (strain 1 F) was performed using a modified protocol described by Sodeik et al.^21^. Briefly, subconfluent BHK-21 cells were infected with RPMI 1640 supplemented with 20 mM Hepes (5 ml/175 cm^2^ flask) containing a low multiplicity of infection (MOI; 0.01). After 1–2 h, 20 ml D10 medium was added and cells were cultivated for 3–4 d, until complete cytopathic effect was observed. Medium was harvested, cell debris was removed via centrifugation at 2575 × *g* and 4 °C for 10 min, and virus containing supernatant was centrifuged at 39,742 × *g* at 4 °C for 2 h. Virus pellets were overlaid with a small volume of PBS at 4 °C overnight. Afterwards, virus pellets were resuspended, aliquoted, and stored at −80 °C until further use.

### Plaque assay

Vero cells, used for viral titration, were cultured in D10 medium (DMEM; Lonza) supplemented with 10% FCS (Merck), 2 mM L-glutamine, 100 U/ml penicillin, and 100 mg/ml streptomycin. Titration of BALF and lung cell suspensions in different dilutions was performed using Vero cells at 100% of confluency. Cells were washed with RPMI 1640 (Lonza, Basel, Switzerland) supplemented with 0.1% BSA (Sigma-Aldrich) and 20 mM Hepes (Lonza) before 200 µl BALF and lung cell suspensions was added. After incubation on a rocking platform for 1 h at room temperature, the inoculum was removed, and 400 µl D10 medium containing 10 µg/ml human IgG (Sigma-Aldrich) were added to each well. Cells were cultured in an incubator for 3 days until visible plaques had formed. Media were discarded, and the cells were fixed with 250 µl of 9% formaldehyde in PBS for 10 min. Afterwards, the formaldehyde solution was removed and 200 µl crystal violet solution (5% crystal violet in ethanol, 1:50 dilution in H2O) was added and incubated for 10 min. Subsequently, wells were washed with water and air-dried. Finally, plaques were counted, and the viral titer was calculated and indicated in PFUs per milliliter.

### Quantitative RT-PCR

Real-time PCR was performed as previously described^22^. Briefly, RNA was extracted from whole tissue by RNeasy mini kit (Qiagen, Venlo, Netherlands). Complementary DNA was reverse transcribed from 1 µg total RNA with Moloney murine leukemia virus reverse transcriptase (Thermo Fisher Scientific) using random hexamer oligonucleotides for priming (Thermo Fisher Scientific). The amplification was performed with a Biorad CFX-Connect Real-time-System (Thermo Fisher Scientific) using the SYBR Green (Eurogentec, Seraing, Belgium) or TaqMan (Thermo Fisher Scientific) detection system. Data were analyzed using the Bio-Rad CFX Manager 3.1 software. The mRNA content for *Cxcl12*, *Ccl2*, *Cxcl9*, and *Ccl21* was normalized to the hypoxanthine–guanine phosphoribosyltransferase (*Hprt*) mRNA for mouse genes. Gene expression was quantified using the ∆∆Ct method. The expression level was arbitrarily set to 1 for one sample from the PBS group and the values for the other samples were calculated relatively to this reference. The sequence of primers is in Supplementary Table 6.

### Cytokine detection

Mouse: Secreted CXCL12 (R&D systems), IFNλ (R&D systems), and IFNα (R&D systems) were measured by ELISA according to the manufacturer’s instructions. Human: Secreted CXCL12 (R&D Systems), IL-3 (R&D Systems), IL-6 (Biolegend), TNF (Biolegend), IFNλ (R&D systems), and IFNα (PBL assay science, Piscataway, NJ, USA) were measured by ELISA according to the manufacturer’s instructions.

### Lung cells stimulation in vitro

Lung cell suspensions from naive mice were cultured in RPMI-1640 GlutaMax supplemented with 10% FCS, 25 mM of Hepes, 1 mM sodium pyruvate, 100U/ml of Penicillin–Streptomycin, and 20 µg/ml of Gentamicin at 37 °C in the presence of 5% CO2. Lung cell suspensions were stimulated in 12-well plates (10^6^ cells/ml) during 24 h by IL-3 (20 ng/ml). Then supernatants were collected for cytokine measurement. CD45^-^ and CD45^+^ cells were purified from lungs of naive mice using CD45 microbeads (Miltenyi Biotec), according to the manufacturer’s instructions.

### Immunohistochemistry

For CXCL12 and EpCAM permanent immunohistochemistry, the staining was performed as previously described^23^. In brief, formalin-fixed, paraffin-embedded lung tissues were deparaffinized by xylene two times for 15 min. The tissue was rehydrated using decreasing concentrations of ethanol (100, 96, 85, and 70%) for 2 min each. Antigen retrieval was performed using Target Retrieval Solution (DakoCytomation) at pH 9.0 at 95 °C for 20 min followed by cooling for 20 min at RT. As a washing buffer between the incubation steps, 1xTBS pH 7.6 was used. The slides were blocked by hydrogen peroxide (7.5%, Sigma-Aldrich), avidin–biotin–block (Vector Laboratories, Burlingame, CA, USA), and 10% donkey normal serum (DNS, Vector Laboratories) in TBS for 10 min. The primary antibodies diluted in 2.5% DNS (rabbit anti-human EpCAM cat no. ab71916, Abcam, 1:300; mouse anti-human CXCL12 cat. no. MAB350, R&D Systems, 1:150) and isotype control antibodies in corresponding concentrations were detected using either the RTU Vectastain Elite ABC Kit anti-mouse/rabbit (for EpCAM and CXCL12; Vector Laboratories) and NovaRed substrate (Vector Laboratories) as a substrate. The slides were counterstained with Gill-III hematoxylin (Merck), dehydrated and mounted with VectaMount permanent mounting medium (Vector Laboratories). The sections were analyzed using a DM6000 B microscope (Leica). NPDview2 software and NanoZoomer 2.0RS software were used to collect immunohistochemistry data and Adobe illustrator CS6 software was used for analysis. The characteristics of the respective patients are detailed in Supplementary Table 2.

### Flow Cytometry

The following antibodies were used for flow cytometric analyses: Mouse: anti-CD317-BV650 (Biolegend), anti-Ly6C-FITC (BD Biosciences), anti-B220-BUV737 (BD Biosciences), anti-CD11c-PerCP Cy5.5 (Biolegend), anti-CD11b-PE CF594 (BD Biosciences), anti-F4/80-BV510 (BD Biosciences), anti-Ly6G-BUV395 (BD Biosciences), anti-SiglecH-Pacific Blue (BD Biosciences), anti-CD45.2-BV786 (BD Biosciences), anti-MHCII-BV711 (BD Biosciences), anti-CD19-BV421 (BD Biosciences), anti-CD3- PerCP Cy5.5 (Biolegend), IgG2a-PE (Biolegend), anti-CD146-FITC (Biolegend), anti-CD31-Pacific Blue (Biolegend), anti-CD326-PE-CF594 (Biolegend), anti-CD326-BV650 (BD Biosciences), and anti-CD123-PE (Biolegend). Human: anti-CD45-Pacific Blue (Biolegend), anti-CD45-BV786 (BD Biosciences), anti-CD303-PerCP Cy5.5 (Biolegend), anti-HLA-DR-BUV395 (BD Biosciences), anti-CD11c-BV711 (BD Biosciences), anti-CD326-PercCP Cy5.5 (Biolegend), anti-CD31-BV711 (BD Biosciences), anti-CD14-BUV737 (BD Biosciences), anti-CD16-BV421 (BD Biosciences), anti-CD11b-BV711 (BD Biosciences), anti-CD15-PE (BD Biosciences), anti-IL-3-PE (BD Biosciences), and anti-IgG1-PE (BD Biosciences). Anti-CXCL12-PE (R&D Systems) and IgG1-PE (R&D Systems) were used for mouse and human. Staining for intracellular cytokines was performed using BD Cytofix/Cytoperm Plus Kit (BD Biosciences). Data were acquired on a Celesta (BD Biosciences) flow cytometer using the BD FACSDiVa™ software v8.0.1.1 and analyzed with FlowJo 10.3.0 (FlowJo LLC, Ashland, OR, USA).

### Human specimen

This prospective, multicentric, observational clinical study was first approved by the local ethics committee on February 1, 2016 (UKER 10_16 B) and modified on April 28, 2020 (UKER 174_20 B). Departments from three additional University Hospitals in Germany participated in this study. Prospective measurements of Interleukin-3 and analysis of patients participating in the trial have been conducted between April 1, 2020 and June 4, 2020. The observational clinical studies were conducted in the medical wards and intensive care units of the (i) University Hospital of Erlangen, Germany; (ii) University Hospital of Essen, Germany; (iii) University Hospital of Giessen, Germany; and (iv) University Hospital of Bonn, Germany. Study patients or their legal designees signed written informed consent. In total, 105 (32 non-severe; 32 severe; 41 recovered) patients positive for SARS-CoV-2 PCR from oral swabs, oral fluid, or BALF were enrolled in this trial. Blood samples were collected at the onset of symptoms (≤24 h), and 1, 2, 3, 4, 5, 6, or 7 days later; or after recovery from SARS-CoV-2 infection (time of recovery = 16 days ± 2 days). Patients with high viral load are patients with CT levels above the median of all patients (Median CT levels: 32.63). Eighteen healthy donors served as controls. Blood: After blood collection, plasma of all study participants was immediately obtained by centrifugation, transferred into cryotubes, and stored at −80 °C until further processing. For flow cytometry analysis, red blood cells were removed by centrifuging blood cells 7 min at 400×*g* without break in Leukosep tube (Greiner, Kremsmünster, Austria). Leukocytes were then stained 20 min at 4 °C in dark and fixed 1 h with BD Cytofix buffer (BD Biosciences). Bronchoalveolarlavage fluid specimen (25 patients): After written informed consent and in agreement with the local ethics review board of the University of Erlangen (UKER no. 4147) the segmental bronchi of patients scheduled for fiber-optic bronchoscopy due to various inflammatory and non-inflammatory conditions were flushed with 100 ml sterile 0.9% saline fluid. Fluid has been obtained and processed for flow cytometric analysis of leukocyte surface markers. In addition, after centrifugation the supernatants have been stored at −80 °C until further processing. Patients with low IL-3 or low CXCL12 are patients with a level of IL-3 or CXCL12 under the mean of all the patients. Patients with high IL-3 or high CXCL12 are patients with a level of IL-3 or CXCL12 above the mean of all the patients. Lung tissue specimen (17 patients): The study was performed at the University of Erlangen in Germany. Patients were selected within the framework of the thoracic surgery board. The patients who underwent surgery and gave their approval were included in this study. The study was performed in agreement with the local ethics review board of the University Hospital of Erlangen (UKER 10_16 B, UKER 339_15 Bc; UKER 56_12B; DRKS-ID: DRKS00005376). Patients’ confidentiality was maintained. The surgery consisted of a wedge resection of the lung or lobectomy. Subsequently, tissue samples were taken from the surgically removed material and transported into the laboratory under standardized conditions (at 4 °C, in Ringer’s solution) for further preparation and analysis. Samples were taken from the non-pathological area from the lung for further analysis. For flow cytometry analysis, separate lung tissue sections were cut into small pieces and subjected to enzymatic digestion with 450  U/ml collagenase I, 125 U/ml collagenase XI, 60 U/ml DNase I, and 60 U/ml hyaluronidase (Sigma-Aldrich, St. Louis, MO) for 1 h at 37 °C while shaking at 750 rpm after which they have been homogenized through a 40 µm nylon mesh for flow cytometric analysis. Total viable cell numbers were obtained using Trypan Blue (Cellgro, Mediatech, Inc, VA).

### Statistics

Results were expressed as mean ± S.E.M. and expressed as identified in legends. For comparing two groups, statistical tests included unpaired, two-tailed parametric *t* tests with Welch’s correction (when Gaussian distribution was assumed), unpaired,two-tailed nonparametric Mann–Whitney tests (when Gaussian distribution was not assumed) or paired, two-tailed parametric *t* tests. *P* values of 0.05 or less were considered to denote significance. For comparing more than two groups, one-way ANOVA was used. Statistics and significant outliers were determined using GraphPad Prism 7.0 software.

### Reporting summary

Further information on research design is available in the Nature Research Reporting Summary linked to this article.

## Supplementary information

Supplementary Information

Reporting Summary

## Data Availability

Data supporting the findings of this manuscript are available from the corresponding authors upon request. Source data are provided with this paper.

## Source data

Source Data
