# Supplementary material for: Interleukin-3 is a predictive marker for severity and outcome during SARS-CoV-2 infections
Source: Nat Commun. 2021 Feb 18;12:1112. doi: 10.1038/s41467-021-21310-4 (PMC7893044; doi:10.1038/s41467-021-21310-4)
Supplement: Supplementary file 1 — Supplementary Information [file 41467_2021_21310_MOESM1_ESM.pdf]

Supplementary information Bénard et al.

Interleukin-3 is a predictive marker for severity and outcome during SARS-CoV-2 infections

Alan Bénard[1*], Anne Jacobsen[1], Maximilian Brunner[1], Christian Krautz[1], Bettina Klösch[1], Izabela Swierzy[1], Elisabeth Naschberger[1], Malgorzata J. Podolska[1], Dina Kouhestani[1], Paul David[1], Torsten Birkholz[2], Ixchel Castellanos[2], Denis Trufa[3], Horia Sirbu[3], Marcel Vetter[4], Andreas E. Kremer[4], Kai Hildner[4], Andreas Hecker[5], Fabian Edinger[5], Matthias Tenbusch[6], Petra Mühl-Zürbes[7], Alexander Steinkasserer[7], Enrico Richter[8], Hendrik Streeck[8], Marc M. Berger[9], Thorsten Brenner[9], Markus A. Weigand[10], Filip K. Swirski[11], Georg Schett[12], Robert Grützmann[1], and Georg F. Weber[1*]

[1] Department of Surgery, Friedrich-Alexander University (FAU) Erlangen-Nürnberg and Universitätsklinikum Erlangen, Erlangen, Germany
[2] Department of Anesthesiology, Friedrich-Alexander University (FAU) Erlangen-Nürnberg and Universitätsklinikum Erlangen, Erlangen, Germany
[3] Department of Thoracic Surgery, Friedrich-Alexander University (FAU) Erlangen-Nürnberg and Universitätsklinikum Erlangen, Erlangen, Germany
[4] Department of Internal Medicine 1 - Gastroenterology, Pneumology and Endocrinology, Friedrich-Alexander University (FAU) Erlangen-Nürnberg and Universitätsklinikum Erlangen, Erlangen, German
[5] Department of Surgery, University Hospital, Giessen, Germany
[6] Institute of Clinical and Molecular Virology, Friedrich-Alexander University (FAU) Erlangen-Nürnberg and Universitätsklinikum Erlangen, Erlangen, Germany
[7] Department of Immune Modulation, Friedrich-Alexander University (FAU) Erlangen-Nürnberg and Universitätsklinikum Erlangen, Erlangen, Germany
[8] Institute of Virology, University Hospital, Bonn, Germany
[9] Department of Anesthesiology and Intensive Care Medicine, University Hospital Essen, University Duisburg-Essen, Essen, Germany
[10] Department of Anesthesiology, Heidelberg University Hospital, Heidelberg, Germany
[11] Center for Systems Biology, Massachusetts General Hospital and Harvard Medical School, Boston, MA, USA
[12] Department of Internal Medicine 3 - Rheumatology and Immunology, Friedrich-Alexander University (FAU) Erlangen-Nürnberg and Universitätsklinikum Erlangen, Erlangen, Germany

[*] Corresponding authors: Georg F. Weber; Alan Bénard

Correspondence to:
Georg F. Weber
Email: georg.weber@uk-erlangen.de
Phone: +49-9131-85-42046

Or

Alan Bénard
Email: alan.benard @uk-erlangen.de
Phone: +49-9131-85-42055

Department of Surgery, Universitätsklinikum Erlangen,Friedrich-Alexander University (FAU) Erlangen-Nürnberg, Erlangen, Germany

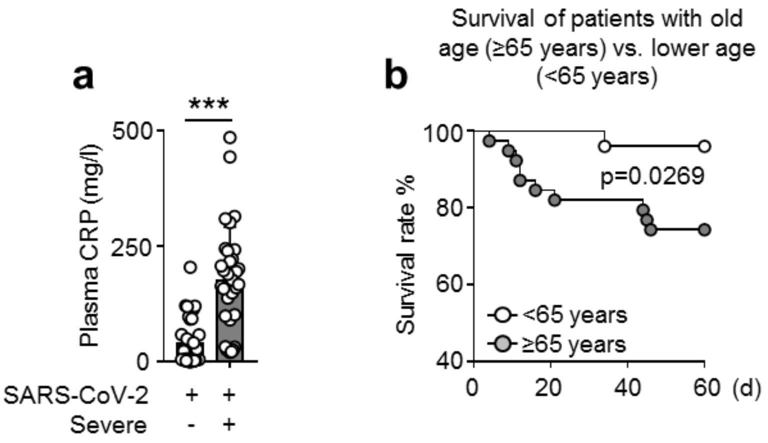

Supplementary Fig. 1. Plasma CRP levels and survival of SARS-CoV-2$^+$ patients with old (≥65 years) vs. lower (<65 years) age. a, Plasma CRP levels in SARS-CoV-2$^+$ patients with severe or non-severe disease. 2-tailed Mann Whitney test. p<0.0001. n=64. **b**, Kaplan-Meier analysis showing the survival of SARS-CoV-2$^+$ patients with old (≥65 years) or lower (<65 years) age. Log-rank test. n=64. Data are mean ± S.E.M., *P < 0.05, **P < 0.01, ***P < 0.001. Source data are provided as a Source Data file.

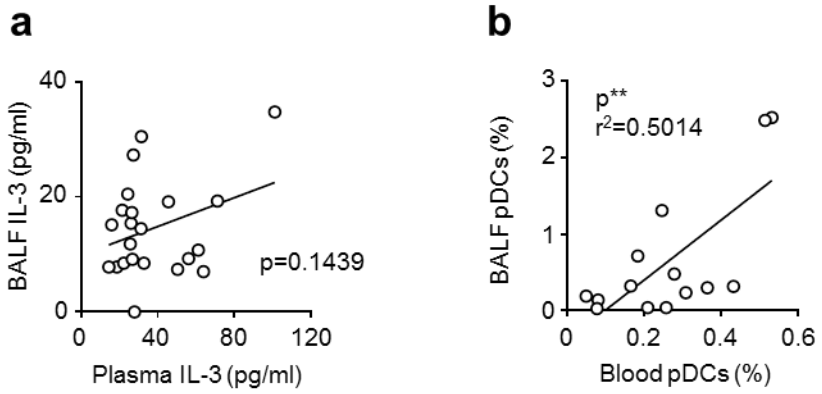

Supplementary Fig. 2. IL-3 levels and pDCs in blood and BALF of patients with pulmonary disease. a, Correlation between plasma IL-3 levels and BALF IL-3 levels in patients with pulmonary disease. Pearson r test. n=22. **b**, Correlation between the percentage of circulating pDCs and the percentage of BALF pDCs in patients with pulmonary diseases. Pearson r test. n=14. Source data are provided as a Source Data file.

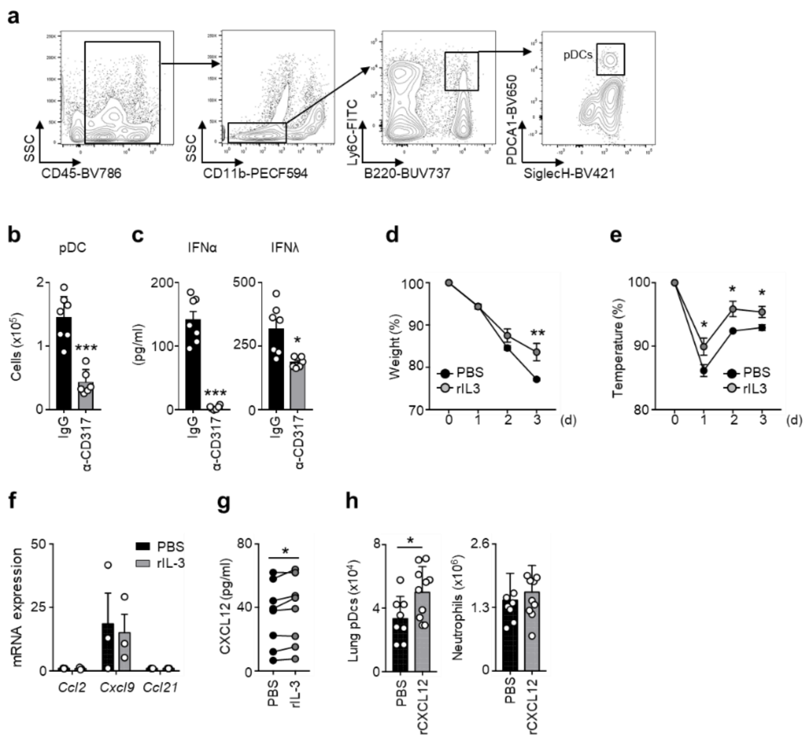

Supplementary Fig. 3. Depletion of pDCs in mice pre-treated with IL-3 induces reduced BALF IFNα and IFNλ levels upon i.n. CpG administration. **a**, Gating strategy used for pDCs. **b-c**, Absolute numbers of pDCs in the lungs (**b**) and levels of IFNα and IFNλ in the BALF (**c**) of naive mice intravenously injected with IgG or anti-CD317 15 h before the injection of rIL-3 and CpG. p<0.0001 (pDCs), p<0.0001 IFNα) and p=0.0107 (IFNλ). n=6-7. **d-e**, Percentage of weight loss (**d**) and body temperature (**e**) in naive WT mice after i.n. infection with $6x10^6$ PFU of HSV-1. (d) p=0.0078 (d3); (e) p=0.0281 (d1), p=0.014 (d2) and p=0.0186 (d3). n=30. **f**, Relative mRNA expression of *Ccl2*, *Cxcl9* and *Ccl21* in the lungs of naive mice 24 h after the i.n. injection of PBS or rIL-3. n=3. **g**, Levels of CXCL12 in the supernatant of lungs cells from naive mice 24 h after *ex vivo* stimulation with or without IL-3. p=0.031. n=8. **h**, Absolute numbers of pDCs or neutrophils in the lungs of naive mice 24 h after the i.n. injection of PBS or rCXCL12. p=0.035. n=8-10 for PBS and n=10 for rCXCL12. Data are mean ± S.E.M., *P < 0.05, **P < 0.01, ***P < 0.001, paired, 2-tailed Student's t test and unpaired, 2-tailed Student's t test using Welch's correction for unequal variances were used. Source data are provided as a Source Data file.

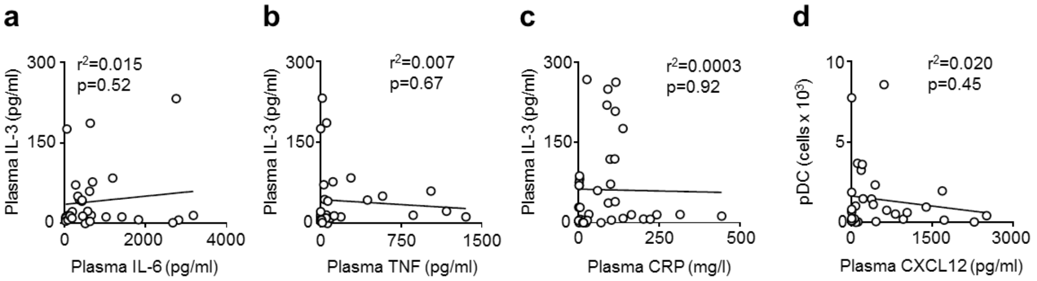

Supplementary Fig. 4. Plasma IL-3 levels do not correlate with plasma IL-6, TNF, CRP levels and circulating pDCs. a-c, Correlation between plasma IL-3 levels and plasma IL-6 (**a**), TNF (**b**) and CRP (**c**) levels in SARS-CoV-2$^+$ patients. Pearson r test. n=31 (a-b) and n=39 (c). **d**, Correlation between plasma CXCL12 levels and the amount of circulating pDCs in SARS-CoV-2$^+$ patients. Pearson r test. n=31. Source data are provided as a Source Data file.

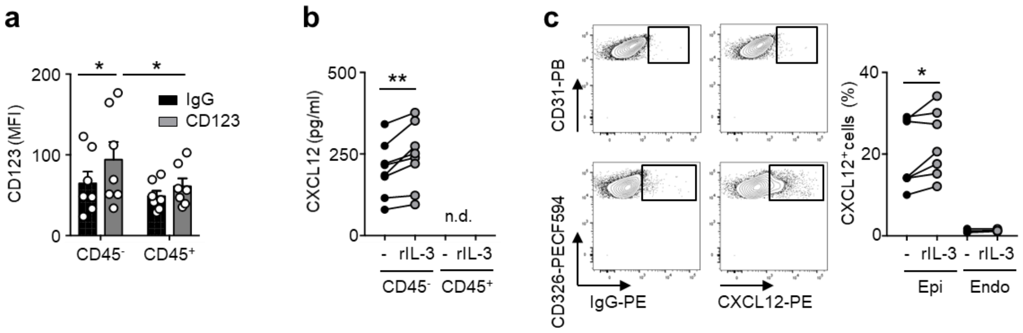

Supplementary Fig. 5. Interleukin-3 induces CXCL12 in lung epithelial cells. a, CD123 expression (MFI) on the surface of $CD45^-$ and $CD45^+$ cells from the lungs of naive mice. p=0.0138 ($CD45^-$) and p=0.0135 ($CD45^+$). n=7. **b**, Level of CXCL12 in the supernatant of $CD45^-$ or $CD45^+$ cells purified from the lungs of naive mice 24 h after *ex vivo* stimulation with or without rIL-3. n=8. **c**, Representative dot plot (left) and percentage (right) of $CXCL12^+$ epithelial or endothelial cells in the lungs of mice 24 h after *ex vivo* stimulation with or without rIL-3. p=0.0319. n=3-7. Data are mean ± S.E.M., *P < 0.05, **P < 0.01, ***P < 0.001, paired, 2-tailed Student's t test and unpaired, 2-tailed Student's t test using Welch's correction for unequal variances were used. Source data are provided as a Source Data file.

Supplementary Table 1: Determing the cutoff threshold of IL-3 plasma levels following SARS-CoV-2 infection based on 60-days survival using the 2-tailed minimal p-value approach (Logrank test; n=64). The optimal cutoff IL-3 plasma level with the lowest p-value is marked grey.

IL-3 plasma level (pg/ml)	p-value (LogRank)	IL-3	N	60-days survival
**50**	0.111	Low	53	79%
		High	11	100%
**30**	0.064	Low	50	78%
		High	14	100%
**25**	0.034	Low	47	77%
		High	17	100%
**20**	**0.027**	**Low**	**46**	**76%**
		**High**	**18**	**100%**
**15**	0.822	Low	39	82%
		High	25	84%
**10**	0.503	Low	35	86%
		High	29	79%
**5**	0.397	Low	24	87%
		High	40	80%

Supplementary Table 2: Multivariate analysis of the impact of different risk factors on mortality following SARS-CoV-2 infection (multivariate logistic regression analysis with univariate Chi-squared test; n=64).

	**Univariate**	**Multivariate**		
	**p-value**	**HR**	**CI**	**p-value**
**Age ≥ 65 years**	**0.042**	-	-	0.157
**Gender**	1.000			
**CRP < 140 mg/l***	**0.001**	0.102	0.019 – 0.552	**0.002**
**IL-3 ≥ 20 pg/ml****	**0.026**	0.000	0.000	**0.026**
**CXCL12 ≥ 10 pg/ml***	0.322			
**Invasive ventilation**	**0.045**	-	-	0.468
**ECMO**	0.134			

*cutoff was determined using p-value approach; bold values are significant (p < 0.05).

**HR (Hazard ratio) and CI (Confidence interval) are "0.000" because no patient died when IL-3 ≥20 pg/ml.

Supplementary Table 3: Risk to die from SARS-CoV-2 according to risk groups

**Risk to die**	**Mortality**	**Mortality**	**OR**	**95%-CI**
**low**	0 / 10 (0%)	1 / 32 (3%)	-	-
**intermediate**	1 / 22 (5%)			
**high**	10 / 32 (31%)	10 / 32 (31%)	14.091	1.680 – 118.218

Low (IL-3 ≥20 pg/ml and age <65 years) and intermediate (IL-3 <20 pg/ml and age <65 years or IL-3 ≥20 pg/ml and age ≥65 years) vs. high (IL-3 <20 pg/ml and age ≥65 years).

Supplementary Table 4: Baseline data of patients scheduled for elective bronchoalveolar lavage.

**Baseline data of the BAL group (n=25)**	
**Demographic data**	
Age, y	58.4 (± 12.4)
Male sex	15 (60%)
**Localization of the BAL**	
Right upper lobe	8 (32%)
Right middle lobe	7 (28%)
Right lower lobe	4 (16%)
Left upper lobe	6 (24%)
Left lower lobe	0 (0%)
**Main diagnosis**	
Primary respiratory	18 (72%)
Non-inflammatory	3 (17%)
NSCLC	2 (67%)
Unclear lung lesion	1 (33%)
Inflammatory	15 (83%)
Bacterial pneumonia	6 (40%)
Undetermined pneumonia	1 (7%)
Undetermined lymphadenopathy	2 (13%)
Undetermined lung fibrosis	2 (13%)
Sarcoid	1 (7%)
COPD	1 (7%)
Tuberculosis	1 (7%)
Systemic sclerosis	1 (7%)
Primary non-respiratory	6 (24%)
Unknown	1 (4%)

Data is presented as the number (%) or the mean (± standard deviation).

Supplementary Table 5: Baseline data of patients scheduled for elective thoracic surgery to obtain lung tissue.

**Baseline data of the lung tissue specimens (n=17)**	
**Demographic data**	
Age, y	62.4 (± 7.4)
Male sex	9 (53%)
**Localization of lung tissue**	
Right upper lobe	7 (41%)
Right middle lobe	0 (0%)
Right lower lobe	4 (24%)
Left upper lobe	2 (12%)
Left lower lobe	4 (24%)
**Main diagnosis**	
Primary respiratory	14 (82%)
Non-inflammatory	12 (86%)
NSCLC	11 (92%)
Chondroharmatoma	1 (8%)
Inflammatory	2 (14%)
IgG4	1 (50%)
Granuloma	1 (50%)
Primary non-respiratory	3 (18%)

Data is presented as the number (%) or the mean (± standard deviation).

Supplementary Table 6: Mouse primer

Name	Forward	Reverse
Hprt	5‘-GTTCTTTGCTGACCTGCTGGAT-3‘	5‘-CCCCGTTGACTGATCATTACAG-3‘
Ccl2	5‘-CCACTCACCTGCTGCTACTCATTC-3‘	5‘-TTCCTTCTTGGGGTCAGCACAGAC-3‘
Cxcl9	5‘-AGCAGTGTGGAGTTCGAGGAAC-3‘	5‘-AGGGATTTGTAGTGGATCGTGC-3‘
Ccl21	5‘-AGAACCTGATGCGCCGC-3‘	5‘-GGCTGTGTCTGTTCAGTTCTCTTG-3‘
Cxcl12	5‘-CTGTGCCCTTCAGATTGTTG-3‘	5‘-TTTCTTCTCTGCGCCCCTT-3‘
