# Supplementary material for: Interleukin-3 is a predictive marker for severity and outcome during SARS-CoV-2 infections
Source: Nat Commun. 2021 Feb 18;12:1112. doi: 10.1038/s41467-021-21310-4 (PMC7893044; doi:10.1038/s41467-021-21310-4)
Supplement: Supplementary file 2 — Reporting Summary [file 41467_2021_21310_MOESM2_ESM.pdf]

nature research

Corresponding author(s): Georg F. Weber, Alan Bénard

Last updated by author(s): Dec 18, 2020

Reporting Summary

Nature Research wishes to improve the reproducibility of the work that we publish. This form provides structure for consistency and transparency in reporting. For further information on Nature Research policies, see Authors & Referees and the Editorial Policy Checklist .

Statistics

For all statistical analyses, confirm that the following items are present in the figure legend, table legend, main text, or Methods section.

n/a	Confirmed	
☐	☒	The exact sample size (*n*) for each experimental group/condition, given as a discrete number and unit of measurement
☐	☒	A statement on whether measurements were taken from distinct samples or whether the same sample was measured repeatedly
☐	☒	The statistical test(s) used AND whether they are one- or two-sided<br>*Only common tests should be described solely by name; describe more complex techniques in the Methods section.*
☐	☒	A description of all covariates tested
☐	☒	A description of any assumptions or corrections, such as tests of normality and adjustment for multiple comparisons
☐	☒	A full description of the statistical parameters including central tendency (e.g. means) or other basic estimates (e.g. regression coefficient) AND variation (e.g. standard deviation) or associated estimates of uncertainty (e.g. confidence intervals)
☐	☒	For null hypothesis testing, the test statistic (e.g. *F*, *t*, *r*) with confidence intervals, effect sizes, degrees of freedom and *P* value noted<br>*Give P values as exact values whenever suitable.*
☒	☐	For Bayesian analysis, information on the choice of priors and Markov chain Monte Carlo settings
☒	☐	For hierarchical and complex designs, identification of the appropriate level for tests and full reporting of outcomes
☒	☐	Estimates of effect sizes (e.g. Cohen's *d*, Pearson's *r*), indicating how they were calculated

Our web collection on statistics for biologists contains articles on many of the points above.

Software and code

Policy information about availability of computer code

Data collection	BD FACSDiVa software v8.0.1.1 was used to collect data from flow cytometry; NPDview2 software and NanoZoomer 2.0RS software were used to collect immunohistochemistry data.
Data analysis	Flow cytometric analyses were performed with the FlowJo analysis software (FlowJo 10.3.0); Statistical analyses were performed with the GraphPad Prism 7.0 software (GraphPad). Immunochemistry analysis were performed with the Adobe illustrator CS6 software. qRT-PCR analysis were performed using the Bio-Rad CFX Manager 3.1 software.

For manuscripts utilizing custom algorithms or software that are central to the research but not yet described in published literature, software must be made available to editors/reviewers. We strongly encourage code deposition in a community repository (e.g. GitHub). See the Nature Research guidelines for submitting code & software for further information.

Data

Policy information about availability of data

All manuscripts must include a data availability statement. This statement should provide the following information, where applicable:

- Accession codes, unique identifiers, or web links for publicly available datasets
- A list of figures that have associated raw data
- A description of any restrictions on data availability

The source data used for Fig.1-5 and Supplementary Figures 1-5 are provided as a Source Data file. Other data supporting the findings of this manuscript are available from the corresponding authors upon request.

Field-specific reporting

Please select the one below that is the best fit for your research. If you are not sure, read the appropriate sections before making your selection.

- [x] Life sciences
- [ ] Behavioural & social sciences
- [ ] Ecological, evolutionary & environmental sciences

For a reference copy of the document with all sections, see nature.com/documents/nr-reporting-summary-flat.pdf

Life sciences study design

All studies must disclose on these points even when the disclosure is negative.

Sample size	Sample size calculations were carried out using group percentages (controls expressing 90% expected value) and variables expressing 20-30% change, which was deemed to be detectable changes using our current imaging and ex vivo analytical methods. Confidence levels were set at 5% with Beta levels established as 50%.
Data exclusions	No data was excluded from the analysis
Replication	All attempts at replication were successful. Findings were replicated in at least three biologically independent samples each.
Randomization	Allocation of animals (mice) into different experimental groups was done in a random manner. No randomisation has been performed for the human data as this is not an interventional study.
Blinding	Investigators were blinded to group allocation during data collection and analysis

Reporting for specific materials, systems and methods

We require information from authors about some types of materials, experimental systems and methods used in many studies. Here, indicate whether each material, system or method listed is relevant to your study. If you are not sure if a list item applies to your research, read the appropriate section before selecting a response.

Materials & experimental systems

n/a	Involved in the study	
	x	Antibodies
x		Eukaryotic cell lines
x		Palaeontology
	x	Animals and other organisms
	x	Human research participants
	x	Clinical data

Methods

n/a	Involved in the study	
x		ChIP-seq
	x	Flow cytometry
x		MRI-based neuroimaging

Antibodies

Antibodies used

The following antibodies were used for flow cytometric analyses at the dilution of 1:200:
Mouse: anti-CD317-BV650 (clone 927; 127019; Biolegend), anti-Ly6C-FITC (clone AL-21; 553104; BD Biosciences), anti-B220-BUV737 (clone RA3-6B2; 612838; BD Biosciences), anti-CD11c-PerCP Cy5.5 (clone HL3; 560584; BD Biosciences), anti-CD11b-PE CF594 (clone M1/70; 553311; BD Biosciences), anti-F4/80-BV510 (clone T45-2342; 743280; BD Biosciences), anti-Ly6G-BUV395 (clone 1A8; 563978; BD Biosciences), anti-SiglecH-Pacific Blue (clone 551; 129609; Biolegend), anti-CD45.2-BV786 (clone 104; 563686; BD Biosciences), anti-MHCII-BV711 (clone M5/114.15.2; 563414; BD Biosciences), anti-CD19-BV421 (clone 1D3; 562701; BD Biosciences), anti-CD3- PerCP Cy5.5 (clone 17A2; 560527; Biolegend), IgG2a-PE (clone RTK2758; 400507; Biolegend), anti-CD146-FITC (clone ME-9F1; 134707; Biolegend), anti-CD31-Pacific Blue (clone 390; 102421; Biolegend), anti-CD326-PE-CF594 (clone G8.8, 118235; Biolegend), anti-CD326-BV650 (clone G8.8; 740559; BD Biosciences), anti-CD123-PE (clone 5B11; 106005; Biolegend).

Human: anti-CD45-Pacific Blue (clone HI30; 304022; Biolegend), anti-CD45-BV786 (clone HI30; 563716; BD Biosciences), anti-CD303-PerCP Cy5.5 (clone 201A; 354210; Biolegend), anti-HLA-DR-BUV395 (clone G46-6; 564040; BD Biosciences), anti-CD11c-BV711 (clone B-ly6; 463130; BD Biosciences), anti-CD326-PercCP Cy5.5 (clone 9C4; 324213; Biolegend), anti-CD31-BV711 (clone WM59; 740777; BD Biosciences), anti-CD14-BUV737 (clone M5E2; 612763; BD Biosciences), anti-CD16-BV421 (clone 3G8; 562874; BD Biosciences), anti-CD11b-BV711 (clone ICRF44; 740771; BD Biosciences), anti-CD15-PE (clone W6D3; 562371; BD Biosciences), anti-IL-3-PE (clone BVD-1F9; 554676; BD Biosciences), anti-IgG1-PE (clone MOPC-21; 555749; BD Biosciences).

Anti-CXC12-PE (clone 79018; IC350P; R&D Systems) and IgG1-PE (clone 11711; IC002P; R&D Systems) were used for mouse and human at the dilution 1:50.

The following antibodies were used for immunohistochemistry analyses: rabbit anti-human EpCAM (cat no. ab71916, Abcam,

	1:300), mouse anti-human CXCL12 (cat. no. MAB350, R&D Systems, 1:150), RTU Vectastain Elite ABC Kit anti-mouse/rabbit (PK7200; Vector Laboratories).<br><br>The following antibody were used for depletion of pDCs: anti-CD317 (clone JF05-1C2.4.1; 130-091-978; Miltenyi) and IgG2b (clone ES26-5E12.4, 130-106-550, Miltenyi). The following antibody were used for depletion of CXCL12: Human/Mouse CXCL12/SDF-1 (clone 79018; MAB350-SP; R&D systems) and mouse IgG1 (clone 11711; MAB002; R&D systems).
Validation	All antibodies have been previously used on murine or human material and are noted as suitable for this purpose by the distributor.

Animals and other organisms

Policy information about studies involving animals; ARRIVE guidelines recommended for reporting animal research

Laboratory animals	Balb/c (WT), C57Bl/6J (WT) (Janvier, Le Genest-Saint-Isle, France) and Cd131-/- (C57Bl/6J background, bred in-house) female mice were used in this study. Majority of the mice were 8-12 weeks old when sacrificed. Mice were breed in animal facility where i) air exchange rate of up to 30 times per hour is possible in the animal rooms; ii) the animals are subject to a 12 hour light / dark rhythm; iii) air humidity is between 45% and 65%; iv) the temperature is 20 ° C and 24 ° C; v) the animals receive autoclaved and autoclavable water; and vi) food and water are ad libitum.
Wild animals	This study did not involve wild animals.
Field-collected samples	This study did not involve samples collected from the field.
Ethics oversight	Oversight had the local animal ethics committee in Dresden and Erlangen. All animal protocols were approved by the animal review committee from the University of Dresden and Erlangen and the local governmental animal committee.

Note that full information on the approval of the study protocol must also be provided in the manuscript.

Human research participants

Policy information about studies involving human research participants

Population characteristics	The study involved patients between 22 and 97 years old and both women and men. Patients came from 4 different german centers (Erlangen, Essen, Giessen and Bonn).
Recruitment	Participants have been continuously recruited on the medical wards and intensive care units of four independent university hospitals in Germany after study participants or their legal designees signed a written informed consent. All participants have been screened for SARS-CoV-2 infections prior to recruiting. To our knowledge, neither self-selection bias or other biases are present in our study.
Ethics oversight	Study oversight was conducted by the local ethics committee at i) University Hospital of Erlangen, Germany; (ii) University Hospital of Essen, Germany; (iii) University Hospital of Giessen, Germany; and (iv) University Hospital of Bonn, Germany.

Note that full information on the approval of the study protocol must also be provided in the manuscript.

Clinical data

Policy information about clinical studies

All manuscripts should comply with the ICMJEguidelines for publication of clinical research and a completedCONSORT checklist must be included with all submissions.

Clinical trial registration	Trial used for obtaining blood samples from SARS2+ patients: UKER 10_16 B and its modified version UKER 174_20 B. Additional trials used for obtaining tissue samples: UKER 10_16 B, UKER 339_15 Bc; UKER 56_12B; UKER 4147; DRKS-ID: DRKS00005376.
Study protocol	Study protocols are either available in the germal trial register (DRKS00005376) or available from the local ethics committee (UKER 10_16 B and its modified version UKER 174_20 B; UKER 10_16 B, UKER 339_15 Bc; UKER 56_12B; UKER 4147).
Data collection	Samples have been continuously collected on the medical wards and intensive care units of the (i) University Hospital of Erlangen, Germany; (ii) University Hospital of Essen, Germany; (iii) University Hospital of Giessen, Germany; and (iv) University Hospital of Bonn, Germany, between April 1st 2020 and June 4th 2020. Samples have been processed and analysed on-site and raw data have been transferred to the university hospital Erlangen for further analysis. Blood samples were collected at the onset of symptoms (≤ 24 hours), and 1, 2, 3, 4, 5, 6, or 7 days later; or after recovery from SARS-CoV-2 infection. For the human bronchoalveolar fluid and tissue samples the time point of collection was immediately after performing the interventional, surgical, or diagnostic procedure.
Outcomes	Primary outcomes: Survival. Secondary outcomes: identification of immunological and clinical parameters for early diagnosis/prognosis of patients with SARS-CoV-2 infection.

Flow Cytometry

Plots

Confirm that:

- [x] The axis labels state the marker and fluorochrome used (e.g. CD4-FITC).
- [x] The axis scales are clearly visible. Include numbers along axes only for bottom left plot of group (a 'group' is an analysis of identical markers).
- [x] All plots are contour plots with outliers or pseudocolor plots.
- [x] A numerical value for number of cells or percentage (with statistics) is provided.

Methodology

Sample preparation	After lungs harvest, single cell suspensions were obtained as follows: perfused lungs were cut in small pieces and subjected to enzymatic digestion with 450 U/ml collagenase I (Sigma Aldrich), 125 U/ml collagenese IX (Sigma Adrich), 60 U/ml hyaluronidase (Sigma Aldrich), 60 U/ml Dnase (Sigma Aldrich) and 20 mM Hepes (Thermo Fisher Scientific, Waltham, MA, USA) for 1 hour at 37° C while shaking. Broncho-alveolar lavage (BAL) was performed by flushing the lungs with 2 × 1 ml of PBS to retrieve the infiltrated and resident leukocytes.
Instrument	FACS LSRII, FACS Celesta
Software	DIVA, FlowJo
Cell population abundance	CD45- and CD45+ cells were purified from lungs of naive WT mice using CD45 microbeads (Miltenyi Biotec). The purity was >97%.
Gating strategy	The pDCs were identified as CD45+ Ly6C high B220 high PDCA1 high SiglecH+.

- [x] Tick this box to confirm that a figure exemplifying the gating strategy is provided in the Supplementary Information.
